# The Cyanobacterial Oxadiazine Nocuolin A Shows Broad-Spectrum Toxicity Against Protozoans and the Nematode *C. elegans*

**DOI:** 10.1007/s00248-025-02507-2

**Published:** 2025-03-04

**Authors:** Ana R. Vieira, Francisco Camacho, Maria L. Sousa, Sara Luelmo, Nuno Santarém, Anabela Cordeiro-da-Silva, Pedro N. Leão

**Affiliations:** 1 Interdisciplinary Centre of Marine and Environmental Research (CIIMAR/CIMAR), University of Porto, Matosinhos, Portugal; 2 Department of Biology and Chemistry, Faculty of Sciences, University of Porto, Porto, Portugal; 3 Institute for Research and Innovation in Health (I3s), University of Porto, Porto, Portugal; 4 Laboratory of Microbiology, Department of Biological Sciences, Faculty of Pharmacy, University of Porto, Porto, Portugal

**Keywords:** Cyanobacteria, Nocuolin A, Protozoa, *C. elegans*, Toxic effect, Amoebae grazing

## Abstract

Cyanobacteria, known to be rich sources of valuable natural products (NPs) with relevant biological properties, are a unique subject to study the interplay between chemistry and ecology. Cultivation of cyanobacteria as isolated strains may only reveal a small fraction of their NPs. In contrast, investigating microbial interactions from an ecological perspective is a particularly fruitful approach to unveil both new chemistry and bioactivity. Cyanobacteria and amoebae are known to co-exist in diverse environments, but the interaction between these organisms has been poorly investigated. Defense strategies against grazer organisms may rely on morphological changes including biofilm formation or increased motility; however, secretion of toxic metabolites seems to be more effective on this regard. Among the most structurally unique cyanobacterial secondary metabolites is nocuolin A, an 1,2,3-oxadiazine metabolite isolated from the cyanobacterial strain *Nodularia* sp. LEGE 06071 that exhibits potent anti-proliferative activity against several human cancer lines, associated with impairment of mitochondrial oxidative phosphorylation. In this work, we show that nocuolin A is toxic against two well-known model amoebae, *Acanthamoeba* and *Dictyostelium*, leading to amoebae encystation and decrease in viability. In addition, in lawn grazing assays, we observed that *Nodularia* sp. LEGE 06071, the producer strain of nocuolin A, was not grazed by amoeba, while a related strain, which does not produce detectable levels of nocuolin A, was. These results support the possible involvement of nocuolin A as a chemical mediator during the interaction between these organisms. Furthermore, we show that this cyanobacterial metabolite also exhibits potent toxicity against other protozoan organisms and a free-living nematode, making it an interesting broad-spectrum scaffold for the development of antiprotozoal or anti-helminthic drugs.

**Supplementary Information:**

The online version contains supplementary material available at 10.1007/s00248-025-02507-2.

## Introduction

Cyanobacteria are photosynthetic bacteria widespread in the environment, that are well-known for their production of secondary metabolites, which can cause toxicity issues in aquatic environments [36] but can also constitute a reservoir of potent bioactivities for industrial and clinical use [3, 18]. As part of screening and biodiscovery programs, a considerable number of cyanobacterial secondary metabolites have been tested for their activity against clinically relevant protozoans or even discovered based on such a feature. These include symplostatin (antimalarial), janadolide and iheyamides A–C (antitrypanosomal), and almiramide B (antileishmanial), and thus cyanobacteria have been considered promising sources for finding antiparasitic agents [20, 32, 37, 45]. Still, the ecological basis for this activity is unclear, as these protozoans do not interact with cyanobacteria during their life cycles. In their different habitats, some cyanobacteria can be grazed by diverse protozoans, such as amoebas [8, 9, 15]. Amoeba-cyanobacteria interactions have been studied to some extent, but the mechanisms that govern them are poorly understood [9, 24]. [25, 26, 43, 49–51, 54]. The studies referred to above have reported that the cyanobacterial strains tested showed different susceptibilities to amoeba grazing: while some cyanobacteria strains can escape predation after being ingested, others are consumed. Cyanobacteria morphology and size have been pointed out as factors responsible for preventing ingestion by amoeba [8, 14, 54], but other studies attribute grazing resistance to yet-unknown compounds [51], more likely to be diffusible secondary metabolites with toxic properties, surface polysaccharides or oligopeptides [43, 49], or volatile organic compounds [30]. Among the bioactive secondary metabolites from cyanobacteria, nocuolin A stands out as it bears a 1,2,3-oxadiazine moiety, a structural feature that is unique among natural products [27, 29]. Nocuolin A was isolated based on its potent anti-proliferative activity against several human cancer lines [53], associated with effects on mitochondrial oxidative phosphorylation [44]. The compound has also been shown to be a potent antifouling agent acting against different fouling species [34]. From a chemical ecology perspective, the demonstrated antifouling activity profile of this compound against microalgae may offer insights into its natural ecological function in cyanobacteria, likely conferring allelopathic properties to the host strains against competing eukaryotic microbial communities [23, 34]. This natural product was initially reported from two different cyanobacterial strains, *Nostoc* sp. CCAP 1453/38 and *Nodularia* sp. HBU26 [53], but was later found to be produced by *Nodularia* sp. LEGE 06071 [27, 44]. These cyanobacteria contain the *noc* locus in their genome (Supplementary Fig. S1), which has been associated with the production of nocuolin A [27, 53], the chlorosphaerolacylates [1], and their hybrids, the nocuolactylates, which seem to be the end-products of the *noc* pathway [10]. To date, these compounds have not been associated with an ecological role.

Here, we initially investigated whether a range of cyanobacterial secondary metabolites could be toxic towards amoeba. While most of the tested compounds did not cause an appreciable effect, nocuolin A was found to be toxic to the model amoebae *Acanthamoeba castellanii* and *Dictyostelium discoideum*. Furthermore, these two amoebas were found to graze a cyanobacterium *Sphaerospermopsis* sp. LEGE 00249 with the *noc* locus that does not produce detectable amounts of nocuolin A, but they did not graze on *Nodularia* sp. LEGE 06071, a producer strain of nocuolin A. We further show that this metabolite is toxic against other protozoans, such as the flagellated parasites *Trypanosom*e and *Leishmania*, causative agents of important human diseases, and against the non-infectious free-living small nematode *Caenorhabditis elegans*. Overall, our results indicate that the unusual metabolite nocuolin A has broad-spectrum and potent toxicity towards a range of protozoans and a nematode, and hint at a possible role in predator-prey interactions between cyanobacteria and amoeba.

## Material and Methods

### Cultivation of Cell Line and Organisms

#### Cyanobacterial Strains

The two cyanobacterial strains used in this work, *Nodularia* sp. LEGE 06071 and *Sphaerospermopsis* sp. LEGE 00249 were obtained from the Blue Biotechnology and Ecotoxicology Culture Collection (LEGE-CC) [35]. These are filamentous cyanobacterial strains that were isolated in different freshwater environments in Portugal. The cyanobacterial strains were cultivated in Z8 medium [19], at 25 °C, with constant aeration, under 14 h/10 h light (10–30 μmol photons s^–1^ m^–2^)/dark cycle.

#### Amoebae Strains

*Acanthamoeba castellanii* (Neff) was kindly provided by Professor Bernard de La Scola, University Aix-Marseille (Marseille, France). *Dictyostelium discoideum* AX2 was requested to the Dicty Stock Center repository (Northwestern University in Chicago, IL, USA). The amoebae strains were maintained aerobically at 25 °C in peptone-yeast-glucose (PYG) medium (20 g protease peptone, 18 g glucose, 2 g yeast extract, 1 g MgSO_4_·_7_H_2_O, 1 g sodium citrate·2H_2_O, 0.02 g Fe(NH_4_)_2_(SO_4_)_2_·_6_H_2_O, 0.06 g CaCl_2_, 0.14 g KH_2_PO_4_, and 0.35 g Na_2_HPO_4_·_7_H_2_O in 1 liter [pH 6.8]; filter sterilized with a 0.22-μm filter, in T25-cm^2^ culture flasks until reach confluent cultures (~10^6^ trophozoites mL^−1^). Sub-cultivation was carried out every 3 days in the same conditions. The concentration and viability of amoebae were determined by the trypan blue exclusion assay, and amoebae were visualized by phase-contrast microscopy with a ×20 objective in an inverted cell culture microscope (Motic AE2000).

#### THP-1 Cell Line

Human leukemia cells, THP-1 (ATCC® TIB­202™), were used as these are the target cells for testing intracellular *Leishmania infantum* activity and for cytotoxicity evaluation [40]. THP-1 cells were cultured in RPMI-1640 medium supplemented with 10% heat-inactivated fetal bovine serum (FBS), 2 mM L-glutamine, 100 UI/mL penicillin/streptomycin, 20 mM HEPES. Cells were maintained in a humidified incubator at 37 °C and 5% CO_2_ by subculture every 3 days in 20 mL of media at a concentration of 1×10^5^ mL^−1^ in a T75-cm^2^ flask. All cell culture reagents were purchased from Lonza-Bioscience.

#### Protozoan Parasites *Leishmania infantum* and *Trypanosoma brucei*

Promastigotes from the *Leishmania infantum* strain (MHOM/MA/67/ITMAP-263) were grown in 5 mL on a T25-cm^2^ culture flasks in Schneider’s insect medium supplemented with 10% heat-inactivated fetal bovine serum (FBS), 200 U/ml penicillin/streptomycin, 6 µg/mL Phenol Red, and 5 mM HEPES. The cultures were maintained in an incubator at 27 °C and diluted to 2×10^5^ mL^−1^ every 5 days. For the assays, the parasites used were equivalent to late/log with two or three days of culture. *Trypanosoma brucei* Lister 427 bloodstream forms were grown in a humidified incubator at 37 °C, 5% CO_2_ in complete HMI-9 medium 36 supplemented with 10% heat-inactivated fetal bovine serum (FBS), and 100 UI/mL penicillin/streptomycin. Parasite maintenance was done in T25-cm^2^ ventilated flasks by sub passage at a concentration of 1×10^4^ mL^−1^ every 2 days. Luciferase-expressing *Leishmania infantum* (MHOM/MA/67/ITMAP-263) axenic amastigotes expressing episomal luciferase were maintained in MAA20 medium at 37 °C, 5% CO_2_ environment with subculture weekly at 1×10^6^ mL^−1^ [41].

#### Nematode *Caenorhabditis elegans*

The *Caenorhabditis elegans* strain used for the study was the wild-type Bristol N2 strain. Animals were grown at 20 °C on nematode growth medium (NGM) agar plates using standard procedures [4]. The plates were seeded with 1 mL of *Escherichia coli* strain OP50 grown overnight at 37 °C and 180 rpm in Luria Broth (LB) media. To obtain the age-synchronized population of eggs, adult gravid hermaphrodites were treated with alkaline hypochlorite solution (0.5 M NaOH, ~2.6 % NaCl_2_) for ~5 min. The animals were washed 3× with M9 buffer, resuspended in S-medium (S-Basal, 10 mM potassium citrate pH 6, 10× trace metals solution (3 mM CaCl_2_, 3 mM MgSO_4_) to the appropriate egg number (25 eggs/15 μL) and transferred into the 96-well plate. The OP50 bacteria were grown overnight at 37 °C and 180 rpm in LB, pelleted by centrifugation, inactivated by three cycles of freeze/thawing, frozen at −80 °C and then resuspended in S-medium supplemented with cholesterol (0.004 mg/mL), penicillin-streptomycin (0.5×), and nystatin (0.5×) (Sigma). The assays that involved *C. elegans* were executed as a service conducted by the Life and Health Sciences Research Institute (ICVS) at the *C. elegans* facility at University of Minho, Portugal.

### Nocuolin A Compound Preparation

Nocuolin A was re-isolated from 40 g lyophilized biomass of cyanobacterial strain *Nodularia* sp. LEGE 06071 (LEGE-CC), using a methanol (MeOH) at room temperature (RT) and 40 °C, followed by a final extraction with dichloromethane (CH_2_Cl_2_), resulting in a crude organic extract of 20 g. The first fractionation was carried out using vacuum-liquid chromatography (VLC) with 20 g of Silica Gel 60 (0.015–0.040, Merk) as the stationary phase. A gradient mobile phase, starting from 10% ethyl acetate (EtOAc) in n-hexanes, progressing to 100% EtOAc, and then to 100% MeOH, was used. These samples were dissolved in MeOH at a concentration of 1 mg mL^−1^ and analyzed by LC/MS on a Thermo Scientific LTQ Orbitrap XL spectrometer, using a gradient from 20% acetonitrile (MeCN) in water to 100% MeCN over 20 min, followed by an isocratic hold at 100% MeCN for 10 min with a flow rate of 1 mL min^−1^. Fractions containing nocuolin A mass features were pooled and subjected to flash chromatography using solvents of increasing polarity. The mobile phase was a mixture of 40% EtOAc in hexanes, progressing to 100% EtOAc, then 50% MeOH, and 100% MeOH. Nocuolin A was isolated using a Waters HPLC system equipped with a 1.525 binary pump and a UV-Vis detector. Separation was achieved with an optimization of the method previously described [44], with a Synergy Fusion-RP column (4 μm, 250 × 10 mm, 80 Å) using a gradient elution from 80 to 100% MeCN over 20 min, followed by 10 min at the same composition, before returning to 80% MeCN in 10 min (flow rate: 2 mL/min). The isolation yielded 16.4 mg of compound. The abundance of nocuolin A was confirmed by LC-MS as previously described, and its purity was assessed using ^1^H nuclear magnetic resonance (NMR) spectroscopy on a BRUKER AVANCE III 400 MHz, 9.4 Tesla spectrometer with a 5-mm cryoprobe. The NMR spectrum was processed using Mnova software v8.0 (Mestrelab Research, S.L.). The compound was found to be pure in excess of 99% by the afore-mentioned ^1^H NMR analysis.

For the toxicity assays using the three amoebae strains, nocuolin A was prepared as a 1 mM working solution in dimethyl sulfoxide (DMSO) (Fisher BioReagents) in glass vials. Stock solutions were prepared, in 100% DMSO, using serial dilution techniques, from the stock (1, 0.5, 0.25, 0.1, and 0.01 µM). Final concentration of DMSO was of 1% in all conditions tested.

### Bioactivity Assays

#### Antiamoebic Activity

The effect of nocuolin A, nocuolactylate A, chlorosphaerolactylate A, and other pure cyanobacterial compounds against *A. castellanii* and *D. discoideum* amoebae species was tested using a colorimetric, miniaturized, and automated plate assay that was designed in this study based on the work of McBride and authors [28], using AlamarBlue (Invitrogen™) cell viability reagent. Briefly, amoebae were grown in T25-cm^2^ culture flasks for 2 to 3 days at 25 °C until confluent cultures and quantified using 10 µL of culture mixed with 10 µL of trypan blue solution 0.4% (Gibco™) on a disposable hemocytometer. Amoebae suspensions were then diluted in PYG medium to obtain a final concentration of 1×10^4^ cells mL^−1^. In non-treated black 96-well plates, in triplicates, 100 µL of PYG medium only was added,100 µL of 1×10^6^ cells mL^−1^ amoebae cells were seed for the control without the compounds, and 99 µL of 1×10^6^ cells mL^−1^ amoebae cells were seed for adding the testing compounds in 1 mM of DMSO (final concentration of 10 µM). The plate was incubated 1 h at 25 °C to allow the cells to seed in the bottom of the wells. Then, 1 µL of testing compounds and 1 µL of the negative control DMSO (final concentration of 1%) and the positive control chlorhexidine digluconate in 10 mM of water (TCI Europe) (final concentration of 100 µM) were added to the plates with overnight incubation at 25 °C. At last, 10 µL of AlamarBlue reagent (10% v/v) was added to the wells, followed by a 24-h incubation at 25 °C. The plate suspensions were observed at the inverted microscope MOTIC AE2000 (Motic), and fluorescence (560 nm excitation and 590 nm emission) was measured using the BioTek Cytation 5 Cell imaging multimode reader (Agilent).

#### Antiparasitic Activity

The efficacy of the compounds against *T. brucei* bloodstream forms and *L. infantum* promastigotes was assessed using a resazurin-based assay, as previously described [5]. Briefly, parasites were introduced to 100 µL of serially diluted compounds at a density of 5×10^3^ cells/mL for *T. brucei* and 5×10^5^ cells/mL for *L. infantum*. A dose-response curve for the anti-trypanosomal drug pentamidine and the anti-leishmanial drug miltefosine was included in all assays. Each well contained a final volume of 200 µL. Each condition was tested in duplicate. After 72 h of incubation under specific conditions for each parasite, 20 µL of a 0.5 mM resazurin solution was added, and the plates were incubated for an additional 4 h. Fluorescence was measured at excitation and emission wavelengths of 544 nm and 590 nm, respectively, using a Synergy 2 Multi-Mode Reader (Biotek). Results were expressed as a percentage of parasite growth inhibition compared to the control (untreated parasites) and represent the average of at least three independent experiments. The effect was evaluated by determining the IC50 value (the concentration required to inhibit growth by 50%), which was calculated using non-linear regression curves with GraphPad Prism version 8.1.1. The activity against *Leishmania infantum* intracellular amastigotes was evaluated following the method described with modifications [40]. Briefly, THP-1 cells were suspended in RPMI complete medium at a density of 1×10^6^ cells/mL. A volume of 100 μL/well was seeded into a 96-well plate and differentiated into macrophages by adding 40 ng/mL of phorbol-myristate 13-acetate (PMA, Sigma) for 24 h, followed by replacement with fresh medium for an additional 24 h. The cells were then infected with *L. infantum* axenic amastigotes expressing episomal luciferase at a macrophage-to-amastigote ratio of 1:10 for 4 h at 37 °C and 5% CO_2_. Non-internalized parasites were washed away, and compounds were added at various concentrations in a final volume of 100 µL. Each condition was tested in quadruplicate. After 72 h of incubation, the medium was replaced with 100 µL of PBS, and 25 µL of Glo-lysis buffer from the Steady-Glo Luciferase Assay System (Promega) was added, and plates were incubated at RT for 10 min. Subsequently, 30 µL of the Steady-Glo reagent (Promega) was added and incubated for 15 min in the dark under the same conditions. A total of 140 µL from each well was transferred to white-bottom 96-well plates, and luminescence intensity was measured using a Synergy 2 Multi-Mode Reader (Biotek). The antileishmanial effect was evaluated by comparison of the non-treated infected cells. The IC50 was determined (for miltefosine) through non-linear regression analysis using GraphPad Prism version 8.1.1. Results represent the average of at least three independent experiments.

#### Anthelmintic Activity

The *C. elegans* Bristol strain N2 was used to screen the toxicity of nocuolin A. The assay was performed in a 96-well plate format, in liquid culture, as previously described [33], [47], [52]. Each well contained a final volume of 60 μL, comprising 20 to 25 animals in egg stage, compounds at different concentrations (0.1,1; 2.5; 5; and 10 μM) and OP50 bacteria to a final optical density of 595 nm of 0.6–0.7. Worms were grown with continuous shaking at 180 rpm at 20 °C for 7 days. The OD_595nm_ was measured daily in the microplate reader (Tecan Infinite 200 Microplate Reader). The effect of compound on *C. elegans* physiology was monitored by the rate at which the *E. coli* food suspension was consumed, as a readout for *C. elegans* growth, survival, or fecundity, therefore being toxicity inferred using this measure. Additionally, visual biometric analysis was performed by the acquisition of images of *C. elegans* culture at days 4, 5, 6, and 7. DMSO 1% (vehicle) and DMSO 5% (toxic condition) controls were used.

### Cytotoxic Effects

The cytotoxic effects of the compounds on THP-1-derived macrophages were assessed using the colorimetric MTT assay (3-(4,5-dimethylthiazol-2-yl)−2,5-diphenyl tetrazolium bromide) in order to test what nocuolin A concentrations are not toxic to the host. Briefly, THP-1 cells differentiated into macrophages using PMA as described above. The cells were then incubated with 100 μL of compounds, ranging from 100 to 12.5 μM after dilution in the RPMI complete medium. Each condition was tested in quadruplicate. After 72 h of incubation at 37 °C with 5% CO_2_, the medium was removed, and 200 μL of a 0.5 mg/mL MTT solution diluted in RPMI was added. The plates were incubated for another 4 h, after which 160 μL of the medium was removed and replaced with the same volume of 2-propanol. Absorbance was measured at 570 nm using a Synergy 2 Multi-Mode Reader (Biotek). Cytotoxicity was evaluated by determining the CC50 value (the drug concentration that reduces the percentage of viable cells by 50%), calculated using non-linear regression analysis with GraphPad Prism version 8.1.1. The results represent the average of at least three independent experiments. For each compound, the selectivity index (SI) was calculated as the ratio between cytotoxicity in THP-1 cells (CC50, 72 h) and activity against parasites (IC50, 72 h).

### Cyst to Trophozoite Culture Assay

In order to test if the removal of nocuolin A compound from the amoebae cultures may lead to the return of the cystic amoebic cells to their active metabolic stage, a cyst to trophozoite culture assay was designed in this study. Briefly, *A. castellanni* was grown in 20 mL of PYG in T25-cm^2^ flasks for 2 to 3 days, and after cell quantification, 1×10^5^ cells mL^−1^ were added to 5 mL PYG medium in T25-cm^2^ culture flasks with 5 µL of nocuolin A at a final concentration of 10 µM, in duplicate. A duplicate control with PYG medium only was used, and duplicate amoebae culture flasks with chlorhexidine digluconate as the positive control and DMSO 1% as the negative control were also used in the assay. The culture flasks were incubated at 25 °C for 24 h statically. Cultures were observed at the inverted microscope for visualization and record of the amoebae cell morphology. Then, the cultures were centrifuged at 7000×*g* for 10 min at room-temperature and the supernatant was removed. The cells were washed twice with 5 mL of sterile phosphate-buffered saline (PBS) 1× medium and centrifuged in the same conditions to remove any possible compounds left. The supernatant was again removed and the cell pellet was suspended in 5 mL of fresh PYG medium and transferred to new T25-cm^2^ culture flasks. The flasks were incubated at 25 °C for 48 h and 72 h statically, and the cultures were again observed at the inverted microscope for visualization and record of the amoebae cell morphology.

### Amoebae Solid Grazing Assay

To test the grazing activity of amoebae on cyanobacteria, two different assays in solid media were conducted and designed in this work based in previous studies [24],Amy T. [25, 26], [30], [43]. For the solid grazing drop assay, briefly, 500 µL of stationary phase cultures of cyanobacterial strains *Nodularia* sp. LEGE 06071 and *Sphaerospermopsis* sp. LEGE 00249 were diluted to 1 mL of fresh Z8 medium in a 24-well plate, and the plate was incubated for 1 week with shaking at 25 °C to allow them to reach an OD_730nm_= 0.7–1.2. The cultures were concentrated by centrifugation, and the pellets suspended in 500 µL of medium. Then, 10 µL drops of the cyanobacterial cultures growing in the 24-well plate were added, in triplicate, onto Z8 agar plates. The agar plates were then incubated at 30 °C for 1 week to allow the cyanobacteria to grow with higher density in the solid medium. On the day of the assay, the amoeba growing in PYG medium, was added at a concentration of 1×10^6^ cells mL^−1^ to Page’s Amoeba Saline Solution (PAS) in T25-cm^2^ culture flasks for 2 h at 25 °C to starve the amoebic cells. The medium recipe for this non-nutrient solution is described in the catalogue of Culture Collection of Algae and Protozoa (CCAP) with the reference QF 09. Lastly, 10 µL drops of *A. castellanii* and *D. discoideum* cultures in PAS medium were added on top of the cyanobacterial culture drops previously grown in the Z8 agar plates. For the control, 10 µL drops of only PAS medium were added to the cyanobacteria, followed by incubation of the agar plates at 25 °C for 2 weeks.

For the solid grazing assay in lawns, 500 µL stationary phase cultures of cyanobacterial strains *Nodularia* sp. LEGE 06071 and *Sphaerospermopsis* sp. LEGE 00249 was plated as lawns onto Z8 agar, and plates were incubated at 30 °C for 1 week to allow for high density growth. Then, 10 µL drops of the amoebae (1×10^6^ cells mL^−1^) were plated on top of the cyanobacterial lawns, and these co-culture plates were incubated at 25° C for 2 to 3 weeks to allow for grazing. Photographs were taken every 3 days to check for the grazing activity phenotype as a yellow clearing area.

### Organic Extraction and LC/MS Analysis

The biomass from the cyanobacterial strains *Nodularia* sp. LEGE 06071 and *Sphaerospermopsis* sp. LEGE 00249 was harvested by scrapping the lawn monocultures and co-cultures (with *A. castellanii* and *D. discoideum*) agar plates after 3 weeks of grazing assay. The biomass was lyophilized and extracted with dichloromethane/methanol CH_2_Cl_2_/MeOH (2:1 v/v) with vigorous vortex at RT. The resulting extracts were filtered with a no. 1 filter paper (Whatman), concentrated using a rotary evaporator (R-210, Buchi) and transferred to pre-weighted vials. The extracts were dissolved in MS grade methanol (VWR Chemicals) to a final concentration of 2 mg mL^−1^ and analyzed by LC-HRMS using an UltiMate3000UHPLC (ThermoFisher Scientific) system, composed of an LPG-3400SD pump, WPS-3000SL autosample, and a VWD-3100UV/vis detector couple to a QExactive Focus Hybrid Quadrupole-Orbitrap mass spectrometer controlled by Q Exactive Focus Tune 2.9 and Xcalibur 4.1 (ThermoFisher Scientific). RMS data were obtained in Full Scan positive and negative mode with a scan range of m/z 150–2000. Cyanobacterial extracts were separated in an UltraCoreTMSuperC18, 2.5-μm particle size, 95Å pore size, and dimensions 75 × 2.1 mm (ACE). The column oven was set to 40 °C. The samples were eluted at 0.40 mL/min with a mobile phase of 0.1% formic acid in 95% water/methanol 5% (eluent A) and in 0.1% formic acid in 95% isopropanol/methanol 5 % (eluent D). The gradient program was as follows: held at 0% D for 1 min, gradient from 0 D to 90% D for 9 min, held at 90% D for 8 min, gradient from 90% D to the initial condition of 0% D for 1 min.

## Results and Discussion

### Effect of Nocuolin A- and noc Locus-Related Compounds on Amoebae Viability

As a starting point of this study, the toxicity of diverse pure cyanobacterial compounds (10 µM); bartolosides A and G [2, 21],desmamides A/B [12],Fischerazoles A-C [10],hierridins B and C [6, 13],nocuolin A [27, 44],and the portoamides a/b [22], was tested against a model strain for host-pathogen interactions, the amoebae *Acanthamoeba castellanii* [38] (Supplementary Fig. S2). It was observed that when the amoebic cells were treated with the disinfectant chlorhexidine digluconate (positive control) and the compound nocuolin A, viability was affected by 80%, showing that this cyanobacterial secondary metabolite has a strong effect against this eukaryotic organism. This response was not observed for all the other compounds tested at this concentration (10 µM), and comparable to the effect caused by the 100 µM chlorhexidine digluconate treatment (Supplementary Fig. S2). For this reason, the bioactivity of nocuolin A (purity degree of >99%, Supplementary Fig. S3) against other protozoa and the interaction of the producer strain of nocuolin A, *Nodularia* sp. LEGE 06071 with amoebae, was further investigated.

The half maximal inhibitory concentration (IC50) was determined from dose-response curves for both the testing compound, nocuolin A, and the positive control, chlorhexidine digluconate, for two model strains of amoebae, *Acanthamoeba castellanii*, found in aquatic environments, and *Dictyostelium discoideum,* a soil amoeba (Supplementary Fig. S4). The IC50 for nocuolin A was higher for *A. castellanii* (1.7 µM) compared with *D. discoideum* (0.1 µM) (Supplementary Fig. S4B, D); the reference compound chlorhexidine diglocunate showed IC50s of 15.6 µM and 1.65 µM, respectively (Supplementary Fig. S4A, C). Hence, nocuolin A was found to have potent toxicity against amoeba, with high nanomolar to low micromolar potency.

**Fig. 1:**
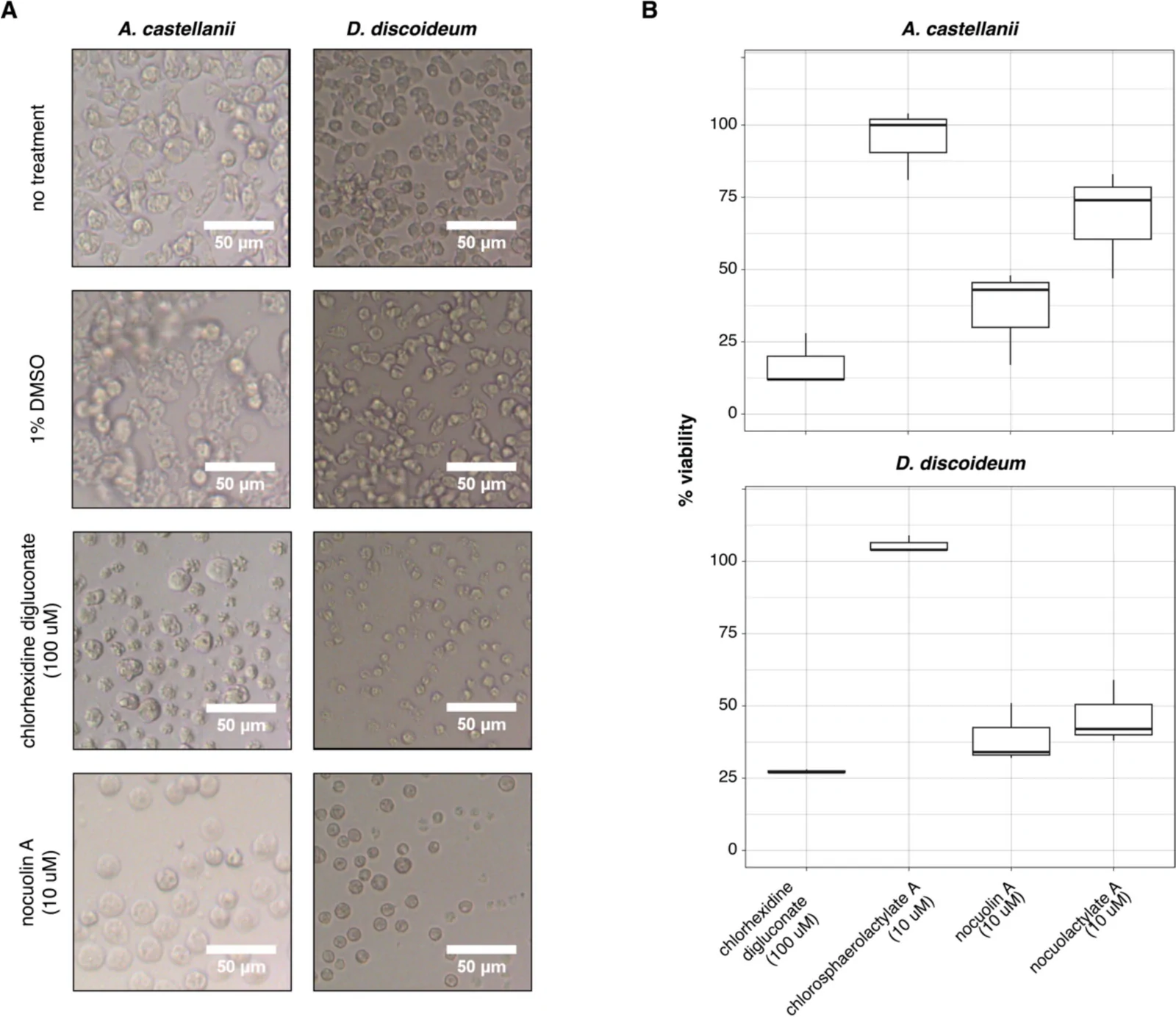
Nocuolin A and *noc*-related compounds are toxic against *A. castellanii* and *D. discoideum* amoebae. **A** Microscopic pictures of *A. castellanii* and *D. discoideum* amoebic cells with no treatment and treated with the negative control DMSO, showing cells in the active trophozoite stage and cells treated with the detergent chlorohexidine digluconate positive control and nocuolin A, showing cells in the cyst dormant stage. **B** The effect of *noc-*related compounds, nocuolin A, nocuolactylate A, and chlorosphaerolactylate A against *A. castellanii* and *D. discoideum* amoebae, showing a decrease in viability when cells are treated with both nocuolin A and nocuolactylate A, as opposite to when cells are treated with chlorosphaerolactylate A. Average of three assays

When in adverse conditions, *Acanthamoeba* and *Dictyostelium* change their active trophozoite form to a dormant double-walled cyst [42]. Microscopic pictures were taken of both amoebae when in the presence of 10 µM of nocuolin A (Fig. 1A), and it was possible to observe that when nocuolin A and chlorhexidine digluconate were added to the amoebae, both strains turned into cysts as compared to the negative control DMSO 1%, that did no encyst (Fig. 1A). It is known that the antiproliferative effects of nocuolin A are associated with effects on mitochondrial oxidative phosphorylation [44], so we hypothesize that nocuolin A compound may act as a mitochondrial toxin, inducing mitochondrial inhibition and, leading to amoebae encystation and decrease in viability. Since the trophozoite-to-dormant cyst transformation is a reversible process, we washed out nocuolin A from the *Acanthamoeba* culture treated with this compound, to test whether the cells could gradually return to the trophozoite form (Supplementary Fig. S5). It was observed that 5 days after washing out both nocuolin A or chlorhexidine digluconate, *A. castellanii* cells fully recovered and were all in the trophozoite form (Supplementary Fig. S5), supporting the direct effect of nocuolin A against amoebae.

We then tested the three compounds that are associated with the *noc* locus of *Nodularia* sp. LEGE 06071 (Supplementary Fig. S1), nocuolin A, chlorosphaerolactylate A, and nocuolactylate A, [1, 10, 27], for their toxicity against *A. castellanii* and *D. discoideum* (Fig. 1B). It was observed that the chlorosphaerolactylates had no effect against amoebae, as previously shown for other organisms, such as fungus and bacteria [16],however, and although not at the same extent as nocuolin A on its own which is highly toxic, the hybrid nocuolactylates decreased approximately 20% of the viability of both *A. castellanii* and *D. discoideum*, likely because the nocuolin A substructure in the noculactylates can still act as a toxic warhead (Fig. 1B).

### Grazing Interaction Between Cyanobacteria and Amoebae

**Fig. 2:**
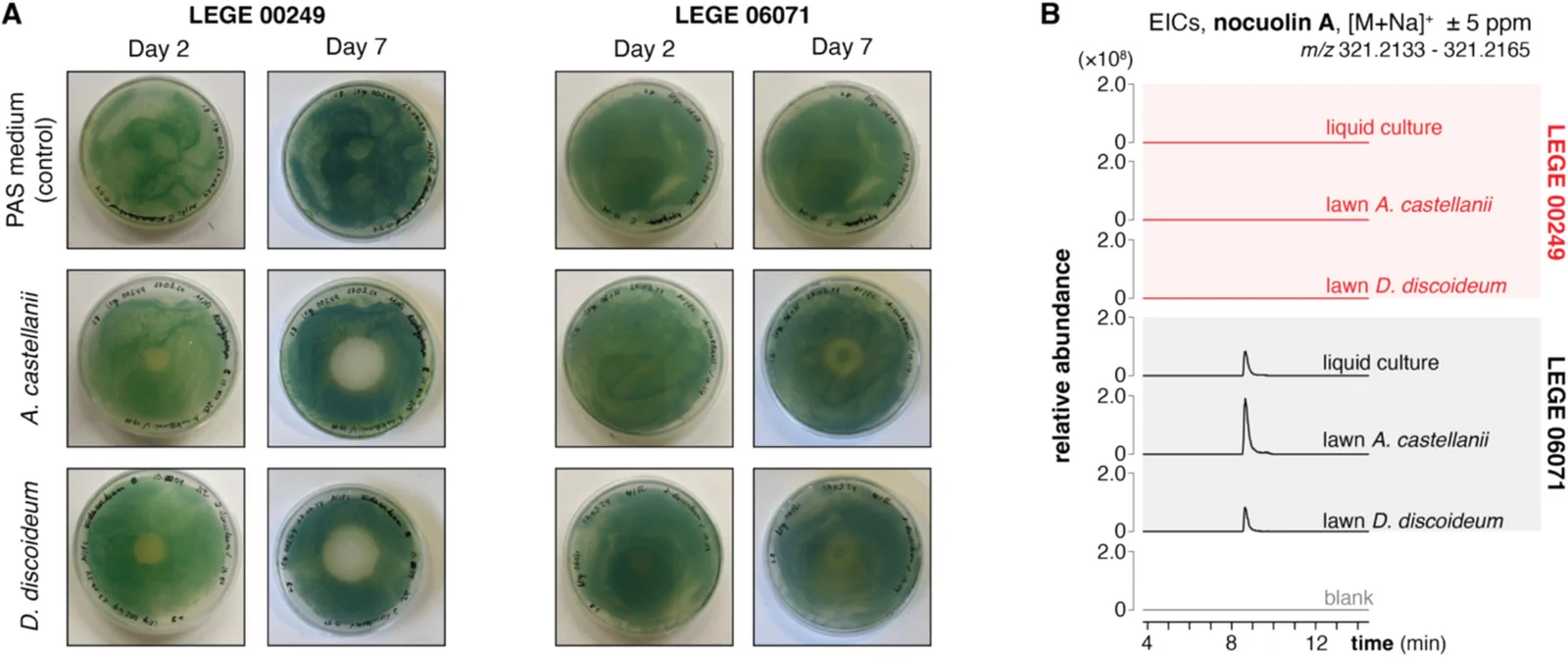
*Nodularia* sp. LEGE 06071, the producer strain of nocuolin A, resists amoebae grazing. **A** Lawns solid grazing assay of cyanobacterial strains LEGE 00249 and LEGE 06071 with *A. castellanii* and *D. discoideum* amoebae overtime, showing grazing resistance for the producer strain of nocuolin A, LEGE 06071 but not for LEGE 00249. Lawns of both cyanobacterial cells with only PAS medium were conducted as the negative control. This was performed as a single assay. **B** LC-HRMS-derived extracted ion chromatograms (EICs) of nocuolin A in the cyanobacterial monocultures and co-cultures of LEGE 06071 and LEGE 00249 with both amoebae, showing that this compound was detected in abundance in the LEGE 06071 lawns, as opposite, to the lawns of LEGE 00249

To investigate whether nocuolin A could have an ecological role as an amoebicide, we studied the ability of the cyanobacterial nocuolin A producer-strain *Nodularia* sp. LEGE 06071 [27], and *Sphaerospermopsis* sp. LEGE 00249 (the producer of the chlorosphaerolactylates [1] and which contains also a *noc* locus, but from which nocuolin A has not been detected/reported), to resist amoebal predation using solid grazing assays. Amoebae cells were deposited onto a solid medium that was previously inoculated with the cyanobacterial strains (either in drops or lawns). Successful grazing resulted in yellow areas devoid of cyanobacteria (Fig. 2A, Supplementary Fig. S6). For the drops grazing assay, 1 week after adding *D. discoideum* to the cyanobacterial suspension drops, it was observed that only the nocuolin A-producing strain, LEGE 06071, was resistant to the grazing (Supplementary Fig. S6A), as opposed to the strain LEGE 00249, which was grazed by these organisms after 5 days (Supplementary Fig. S6B). Unexpectedly, both cyanobacterial strains LEGE 06071 and LEGE 00249 showed resistance to *A. castellanii* in the solid drop assay (data not shown). In the lawns grazing assay, it was observed that when both *A. castellanii* and *D. discoideum* amoebae were added to the LEGE 06071 lawn, this cyanobacterial strain was initially grazed but could then recover and start re-growing in the initial grazing spot, as opposite to the strain LEGE 00249 that after being grazed could not recover (Fig. 2A). To confirm that nocuolin A was being produced during the interaction between *Nodularia* sp. LEGE 06071 and amoebae, the lawns were analyzed for the presence of the metabolite. Organic extraction (2:1 CH_2_Cl_2_/MeOH, v/v) of scrapped biomass from the solid grazing lawns of co-cultures of LEGE 06071 or LEGE 00249 with *A. castellanii* and *D. discoideum* versus the monocultures of cyanobacterial strains alone, followed by LC/MS analysis, was performed to detect levels of nocuolin A, as well as those of other *noc* locus compounds (Fig. 2B, Supplementary Fig. S7). As expected, nocuolin A, was detected in high abundance (~10^8^ ion counts) on the mono-cultures and co-cultures of LEGE 06071 with both amoebae (Fig. 2B). Still, abundance of the metabolite was not noticeable higher in co-cultures with amoeba vs. monocultures. The chlorosphaerolactylates were detected in high abundance for strain LEGE 00249 (~10^8^ ion counts) and in low abundance for LEGE 06071 (~10^5^ ion counts, Supplementary Fig. S7). Again, no evident differences were observed in the monocultures and co-cultures of LEGE 00249. Concerning the hybrid nocuolactylates, these were detected in high and similar abundance only in the monocultures and co-cultures of LEGE 06071 (~10^7^ ion counts, Supplementary Fig. S7). Unlike what was previously reported, nocuolin A was detected in the monocultures and co-cultures of LEGE 00249, but it was detected in very low abundance, three orders of magnitude lower than in LEGE 06071 (Fig. 2B). Taken together, our results show that the nocuolin A-producing strain *Nodularia* sp. LEGE 06071 resists amoebae predation, and suggest a role of nocuolin A as an ecologically relevant toxic compound or deterrent. Nevertheless, the levels of compound in the cells do not seem to respond to the challenge with amoeba cells. We could not generate a mutant strain of *Nodularia* sp. LEGE 06071 unable to produce nocuolin A, which would provide direct evidence for its involvement in the observed amoeba behavior. Genetic engineering in non-model cyanobacterial strains has several limitations, since for example, only a few cyanobacteria are naturally competent [31], [46].

### Activity of nocuolin A against trypanosomatids protozoa parasites and nematode *C. elegans*

*Leishmania* spp. and *Trypanosoma* spp. are parasitic flagellate protozoa that are causative agents of human diseases, Leishmaniasis and Trypanosomiasis (also known as sleeping sickness), respectively, that are transmitted to humans through the bites of infected flies [7, 39]. Currently, there is no human vaccine available for both these trypanosomatids, and the treatment options available present significant limitations related to activity, toxicity, and the emergence of resistant parasites. Therefore, new drug targets, vaccine candidates, and improved diagnostic approaches are needed to tackle these diseases [7]. Because of the observed antiamoebic activity demonstrated by nocuolin A, we decided to test this compound against these two protozoan parasites, *Trypanosoma brucei* and *Leishmania infantum* (Table 1, Supplementary Fig. S8).

**Table 1:** Half-maximal inhibitory concentration (IC50) and 95% confidence interval (95% CI) of nocuolin A, miltefosine, and pentamidine against promastigotes and amastigotes forms of *Leishmania infantum* and *Trypanosoma brucei* parasites and cytotoxicity to THP-1 cells. Values were obtained from at least three independent screening assays

	*Trypanossoma brucei*	*Leishmania infantum*		THP-1 Cells
	IC50 (μM) 95% CI (profile likelihood)	IC50 (μM) 95% CI (profile likelihood)		CC50 (μM) 95% CI (profile likelihood)
		Promastigotes	Amastigotes	
Nocuolin A	3.99 (3.72 to 4.25)	0.21 (0.18 to 0.24)	67.0 ± 17.7*	2.13 (1.40 to 3.15)
Pentamidine	0.003 (0.002 to 0.003)	NT	NT	NT
Miltefosine	NT	8.37 (7.02 to 9.91)	2.12 (1.39 to 3.15)	NT

*NT*, not tested

*Representation of mean percentage growth inhibition ± standard deviation in a single dose at 1μM

The molecule presents activity against *T. brucei* within a low micromolar range, with an IC50 of 3.99 µM (Table 1, Supplementary Fig. 8A). Nocuolin A was also active against *L. infantum* promastigote forms presenting sub micromolar potency with an IC50 of 0.21 µM (Table 1, Supplementary Fig. 8B). Considering that *L. infantum* is an intracellular pathogen, the activity of nocuolin A was also tested in a more relevant intracellular *L. infantum* amastigote infection model using THP-1 as host cells [40]. General toxicity was evaluated in THP-1 cells, the target cell for testing intracellular *L. infantum* activity, in which the calculated CC50 (50% cytotoxic concentration) of the compound in these macrophage cells was 2.1 µM (Table 1, Supplementary Fig. 8C). This limited the capacity to perform activity testing in intracellular amastigotes at higher concentrations. At 1 µM, nocuolin A showed 67% inhibition (Table 1). It was not possible to test in the intracellular model at higher concentrations because the calculated CC50 in THP-1 cells was 2.13 μM.

Miltefosine and pentamidine were tested as reference drugs for leishmaniasis and trypanosomiasis, respectively, in each correspondent assay (Table 1, Supplementary Fig. 8D-F). Miltefosine presented an IC50 of 8.4 μM against promastigotes forms of *L. infantum* and pentamidine presented and IC50 of 0.003 μM against *T. brucei* parasite (Table 1, Supplementary Fig. 8D, F). The control miltefosine in *L. infantum* amastigote forms presented an IC50 of 2.12 μM (Table 1, Supplementary Fig. 8E). Considering these results, there seems to be a reduced therapeutic margin for the molecules in these pathogens as predicted selectivity indexes are low; however, medicinal chemistry efforts might improve the nocuolin A scaffold as observed previously [48].

Parasitic nematodes infect many species of animals, including humans, and are able to parasitize plants which is a global problem for agriculture [17]. Despite *Caenorhabditis elegans* being a non-hazardous, non-infectious, non-pathogenic, non-parasitic organism, it has been used as a model organism for mode of action studies for anthelmintics and nematicides, since the parasitic nematodes are very difficult to work with, requiring passage through their host for maintenance of their parasitic life-cycle [17].

Anthelmintic drugs are still relatively few in number and are losing their effectiveness because nematode strains with resistance are emerging, and many of them are limited in their usefulness because of a narrow spectrum of action, safety, high cost, or impractical delivery systems [11]. Although ivermectin has been successful in parasite treatment in humans, it has led to decreased motivation for the discovery of other anthelmintic drugs, and in the last 5 years, resistance to ivermectin has been emerging [11]. There is an urgent need to develop novel anthelmintics in view of the increasing threat to livestock and humans from anthelmintic-resistant strains of parasites [11].

**Fig. 3:**
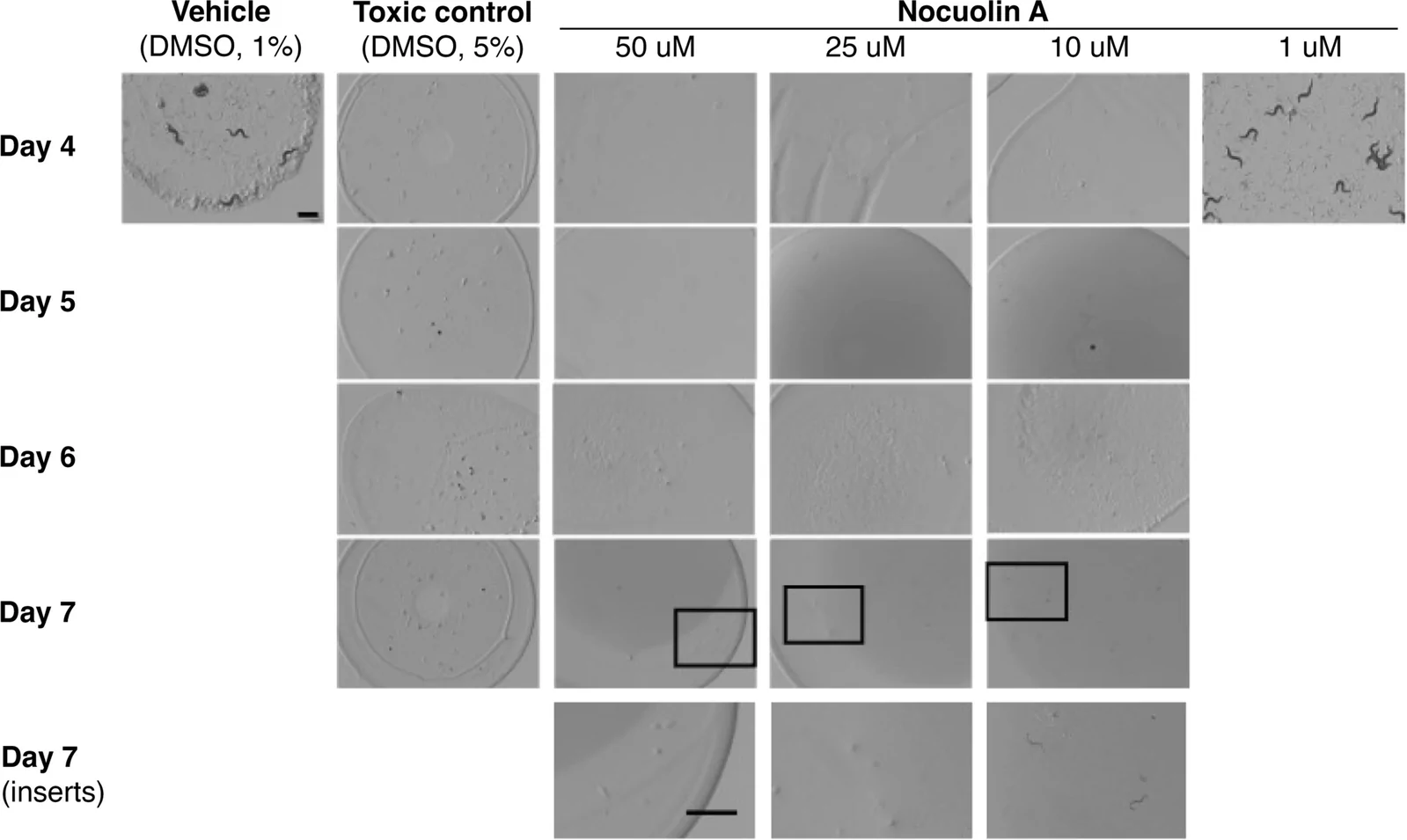
Visual inspection of *C. elegans* treated with nocuolin A. Treatment impact was visually evaluated from day 4 to day 7 of the assay. A strong impact on *C. elegans* development was observed when animals were treated with nocuolin A at or above 10 μM, concentration at which animals were unable to reach adulthood at day 7 of the assay. Animals treated with 1 μM of drug and below presented normal development, with adult worms and progeny being detected at day 4. This assay was performed in triplicate. Scale bar—1 mm

To evaluate the bioactivity spectrum of nocuolin A, the toxicity of the compound was also tested against the free-living nematode *C. elegans*, by performing a food clearance assay in this organism (Fig. 3, Supplementary Fig. S9). For this assay, 1% DMSO corresponding to the drug vehicle was used as the negative control, and DMSO at 5% was used as a positive control since this concentration is known to be toxic to *C. elegans.* Concentrations of nocuolin A at 50 μM, 25 μM, 10 μM, and 5 μM were considered toxic for *C. elegans*; concentrations below 2.5 µM and the negative control, DMSO 1%, had no impact against this organism (Supplementary Fig. S9). Additionally, visual biometric analysis was performed through the acquisition of images of *C. elegans* in culture over time (Fig. 3). The development of *C. elegans* was severely impaired when treated with nocuolin A at 10 μM, with animals being unable to reach adulthood at day 7 of the assay. A moderate impact was observed in the development and progeny at 5 μM and 2.5 μM with no progeny presence at day 4. Animals treated with 1 μM of drug and below presented normal development (Fig. 3).

Therefore, and as concluded for the parasitic protozans, the unusual scaffold of nocuolin A can represent an interesting starting point for anthelmintic drug development.

## Conclusion

A variety of cyanobacterial secondary metabolites have been found to be highly toxic to potential foragers of cyanobacteria. Still, assigning a natural role of defense against foraging to such compounds is not always straightforward. In this study, we have explored the bioactivity of nocuolin A, a cyanobacterial natural product with known cytotoxic and antifouling activity, against a selection of target protozoan organisms, some of which may encounter cyanobacteria in the natural setting (ecologically relevant), and some that have clinical relevance. We observed broad-spectrum activity of nocuolin A against several protozoans, including the amoebae grazers *Acanthamoebae* spp. and *D. discoideum*, and the flagellated parasites *L. infantum* and *T. brucei*. Given its broad scope of activity, we further tested the compound against the free-living nematode *C. elegans*, to which it was also toxic*.* Our data suggests that nocuolin A may act as a chemical-mediator in the interactions between the cyanobacterium *Nodularia* sp. LEGE 06071 and amoeba, namely by having a defensive or deterrent role. Given its broad toxicity, it may act as defense molecule against other predators, but this was not tested in the present study. Although cyanobacteria and amoeba co-exist in a variety of environments, their interactions have been poorly studied, and this is the first study that provides evidence towards an anti-amoebic role for a cyanobacterial secondary metabolite. Furthermore, our findings indicate that nocuolin A might serve as a privileged scaffold for antiprotozoan or anthelmintic drug development.

## Supplementary Information

Below is the link to the electronic supplementary material.

Supplementary file1 (PDF 20758 KB)

## Data Availability

No datasets were generated or analysed during the current study.
