# Supplementary material for: The Cyanobacterial Oxadiazine Nocuolin A Shows Broad-Spectrum Toxicity Against Protozoans and the Nematode C. elegans
Source: Microb Ecol. 2025 Mar 4;88:9. doi: 10.1007/s00248-025-02507-2 (PMC11880066; doi:10.1007/s00248-025-02507-2)
Supplement: Supplementary file 1 [file 248_2025_2507_MOESM1_ESM.pdf]

Supplementary information

The cyanobacterial oxadiazine nocuolin A shows broad-spectrum toxicity against protozoans and the nematode *C. elegans*

Ana R. Vieira[1], Francisco Camacho[1,2], Maria L. Sousa[1], Sara Luelmo[3], Nuno Santarém[3,4], Anabela Cordeiro-da-Silva[3,4], Pedro N. Leão[1,*]

[1]Interdisciplinary Centre of Marine and Environmental Research (CIIMAR/CIMAR), University of Porto, Matosinhos, Portugal

[2]Department of Biology and Chemistry, Faculty of Sciences, University of Porto, Porto, Portugal

[3]Institute for Research and Innovation in Health (i3S), University of Porto, Porto, Portugal

[4]Laboratory of Microbiology, Department of Biological Sciences, Faculty of Pharmacy, University of Porto, Porto, Portugal

Supplementary Figure S1. Schematic highlighting *noc* locus and its proposed relationship with nocuolin A, chlorosphaerolactylate A and the nocuolactylate A in the cyanobacterial strain *Nodularia* sp. LEGE 06071.

Supplementary Figure S2. The cyanobacterial compound nocuolin A affects *Acanthamoeba castellanii* viability.

Supplementary Figure S3. 1H NMR (400 MHz) spectra of nocuolin A isolation in chloroform-d.

Supplementary Figure S4. Dose response curves used to generate the IC50 of chlorhexidine digluconate and nocuolin A in *Acanthamoeba castellanii* and *Dictyostelium discoideum*.

Supplementary Figure S5. Microscope pictures of *A. castellanii* cells after treatment with chlorhexidine digluconate and nocuolin A before and after its removal overtime.

Supplementary Figure S6: *Nodularia* sp. LEGE 06071 is resistant to *D. discoideum* grazing in a solid medium drop grazing assay.

Supplementary Figure S7: Detection of nocuolactylates and chlorosphaerolactylates in the lawns of the monocultures and co-cultures of *Nodularia* sp. LEGE 06071 and *Sphaerospermopsis* sp. LEGE 00249 with amoebae.

Supplementary Figure S8: Dose response curves used to generate the IC50 of nocuolin A and the control compounds miltefosine and pentamidine in *Trypanosoma brucei*, *Leishmania infantum* and THP-1 cells.

Supplementary Figure S9: Nocuolin A toxicity evaluation using *C. elegans*.

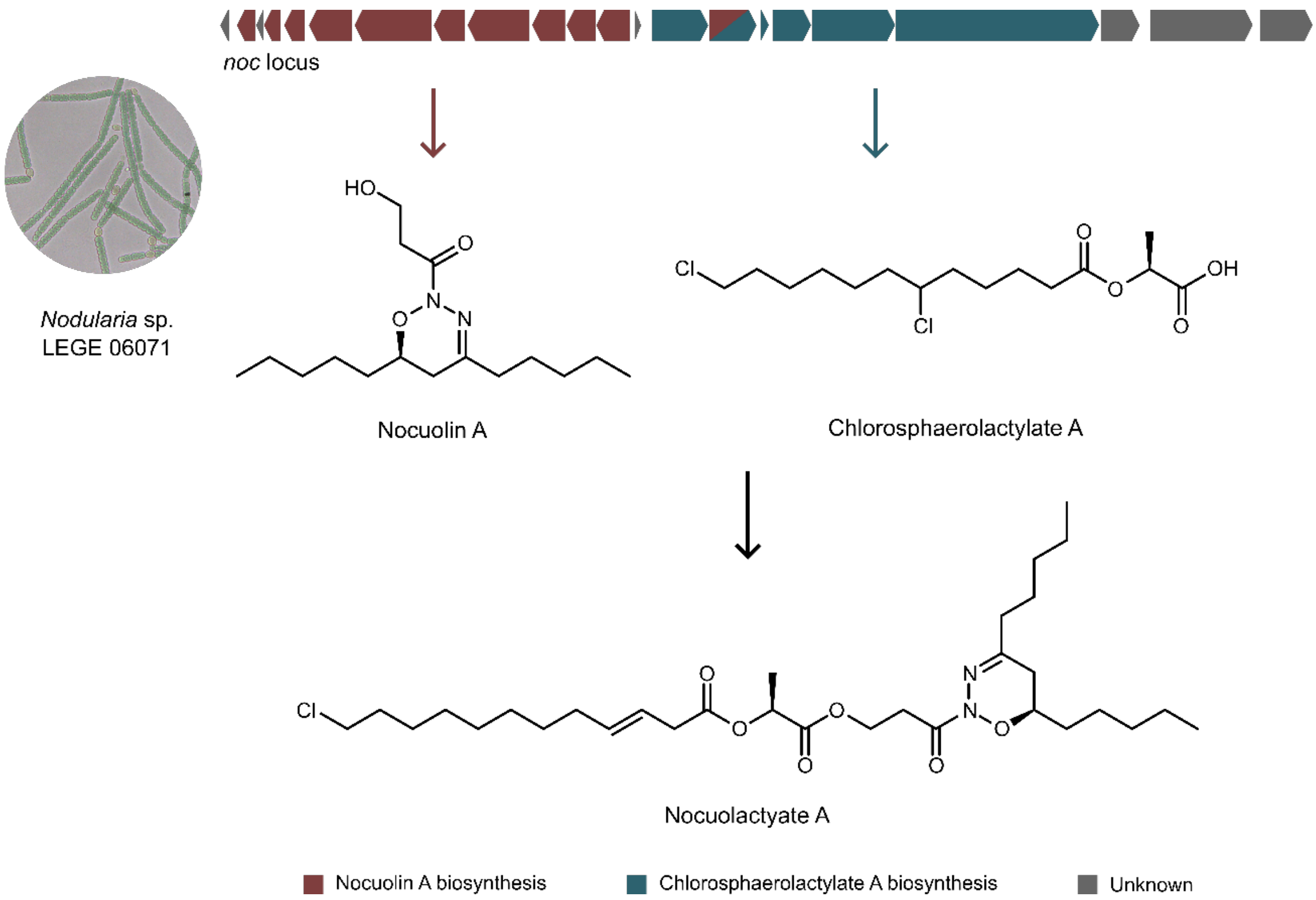

Supplementary Fig. S1. Schematic highlighting *noc* locus and its proposed relationship with nocuolin A, chlorosphaerolactylate A and the nocuolactylate A in the cyanobacterial strain *Nodularia* sp. LEGE 06071. A portion of the ≈40 kb BGC putatively assigned to nocuolin A (in red), and another portion putatively assigned to the chlorosphaerolactylates (in green). The nocuolactylates could be products of both red and green represented BGC portion, which co-localize in cyanobacterial genomes, however, further evidence is required to unequivocally connect *noc* genes to these metabolites (Figueiredo *et al*., 2021; Martins *et al*.,2022). The cyanobacterial strain *Sphaerospermopsis* sp. LEGE 00249 also contain the *noc* locus but only the chlorosphaerolactylates were reported for this strain.

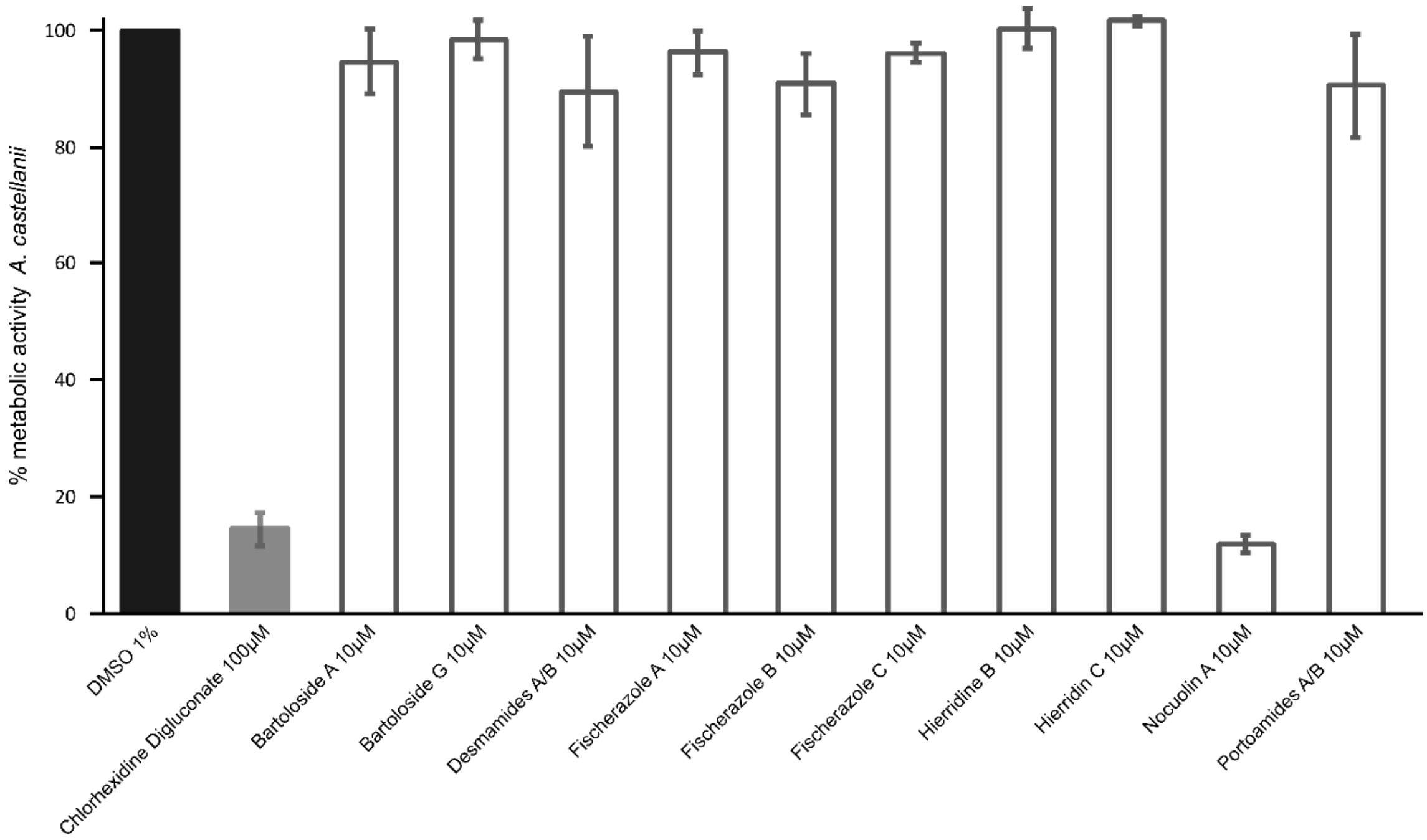

Supplementary Fig. S2 The cyanobacterial compound nocuolin A affects *Acanthamoeba castellanii* viability. The toxicity of diverse cyanobacterial pure compounds (10 µM) was tested against *A. castellanii*. The detergent chlorhexidine digluconate was used as the positive control. Average of three assays. Black bar represents *A. castellanii* cells treated with DMSO 1 % (negative control); grey bar represents *A. castellanii* cells treated with chlorhexidine digluconate (positive control) and white bars represent *A. castellanii* cells treated with the diverse cyanobacterial compounds.

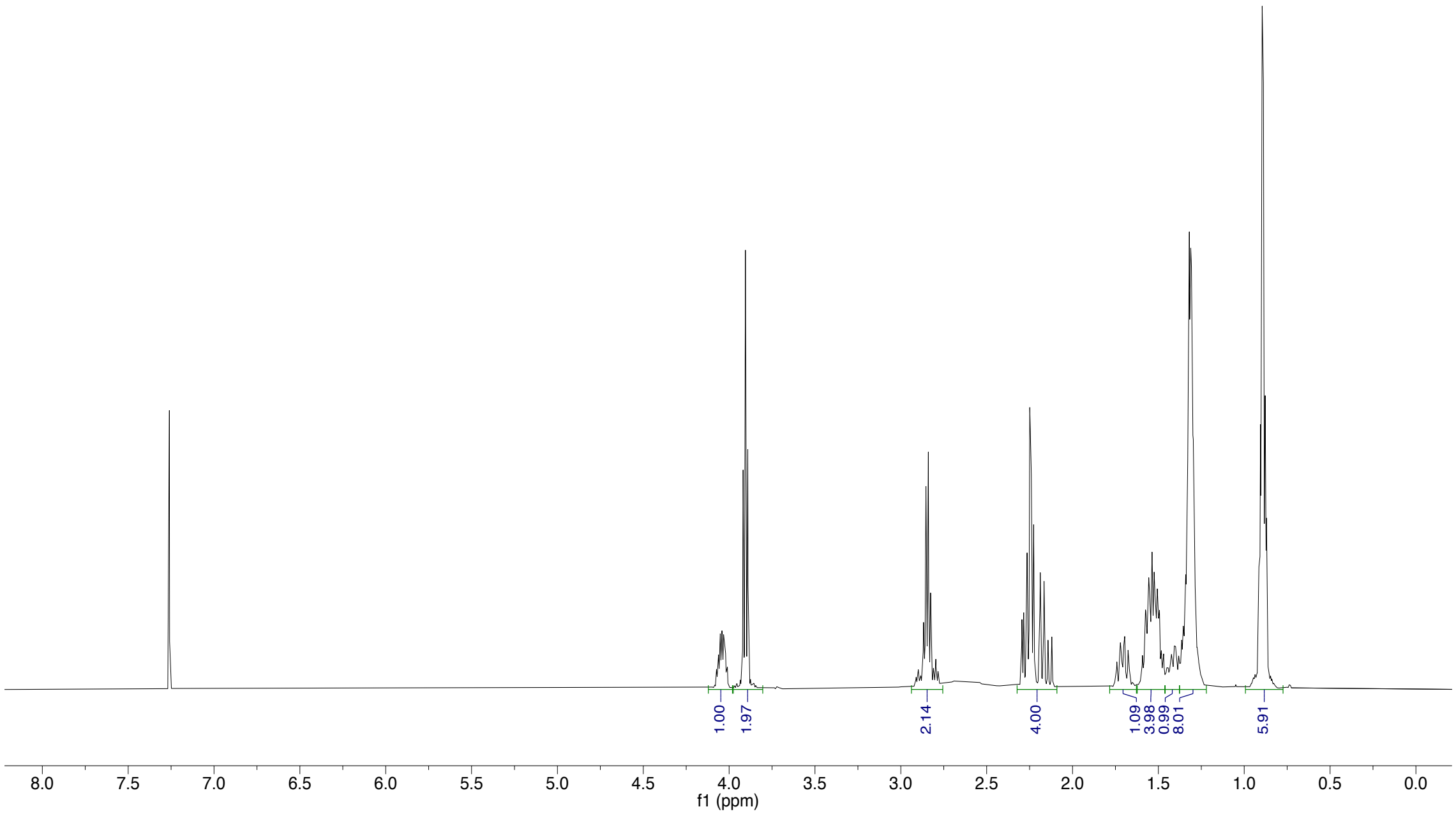

Supplementary Fig. S3. ^{1}H NMR (400 MHz, $CDCl_3$) spectrum of nocuolin A (> 99% purity) used in this study. The non-integrated broad signal at ~2.75 ppm corresponds to the exchangeable alcohol proton.

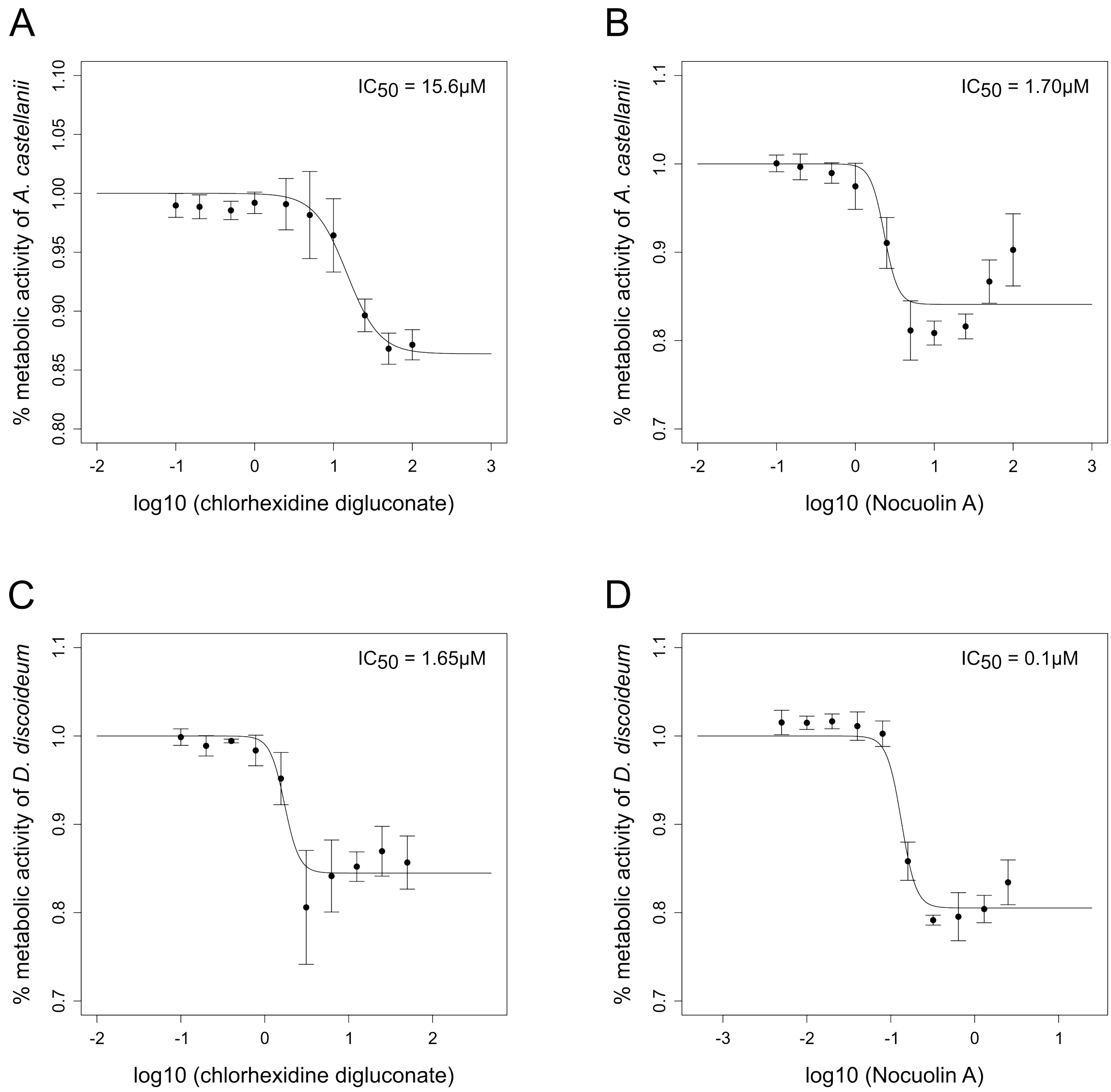

Supplementary Fig. S4: Dose response curves used to generate the IC50 of A) chlorhexidine digluconate and B) nocuolin A in *Acanthamoeba castellanii* and, C) chlorhexidine digluconate and D) nocuolin A in *Dictyostelium discoideum*. Average of three assays. The IC50 was 15.6 µM and 1.70 µM for chlorhexidine diglocunate and nocuolin A in *A. castellanii* and 1.65 µM and 0.1 µM, respectively for *D. discoideum*.

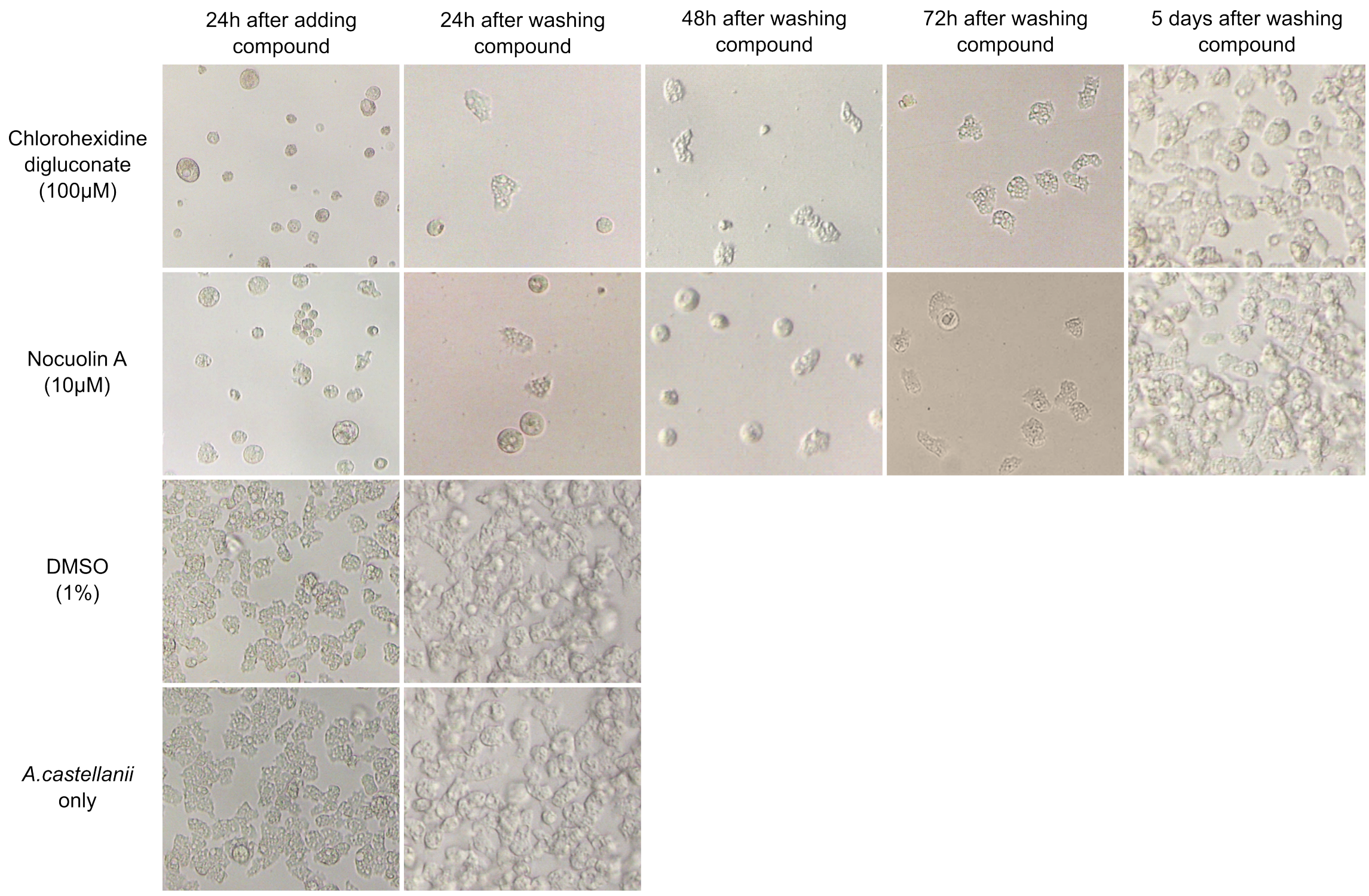

Supplementary Fig. S5: Microscope pictures of *A. castellanii* cells after treatment with chlorhexidine digluconate and nocuolin A before and after its removal overtime. Amoeba cells encysted after adding the positive control chlorhexidine diglocunate and nocuolin A, but could recover after a few days of washing out these compounds. *A. castellanii* cells without treatment and treated with DMSO 1 % were used as negative controls and are observed as active trophozoites.

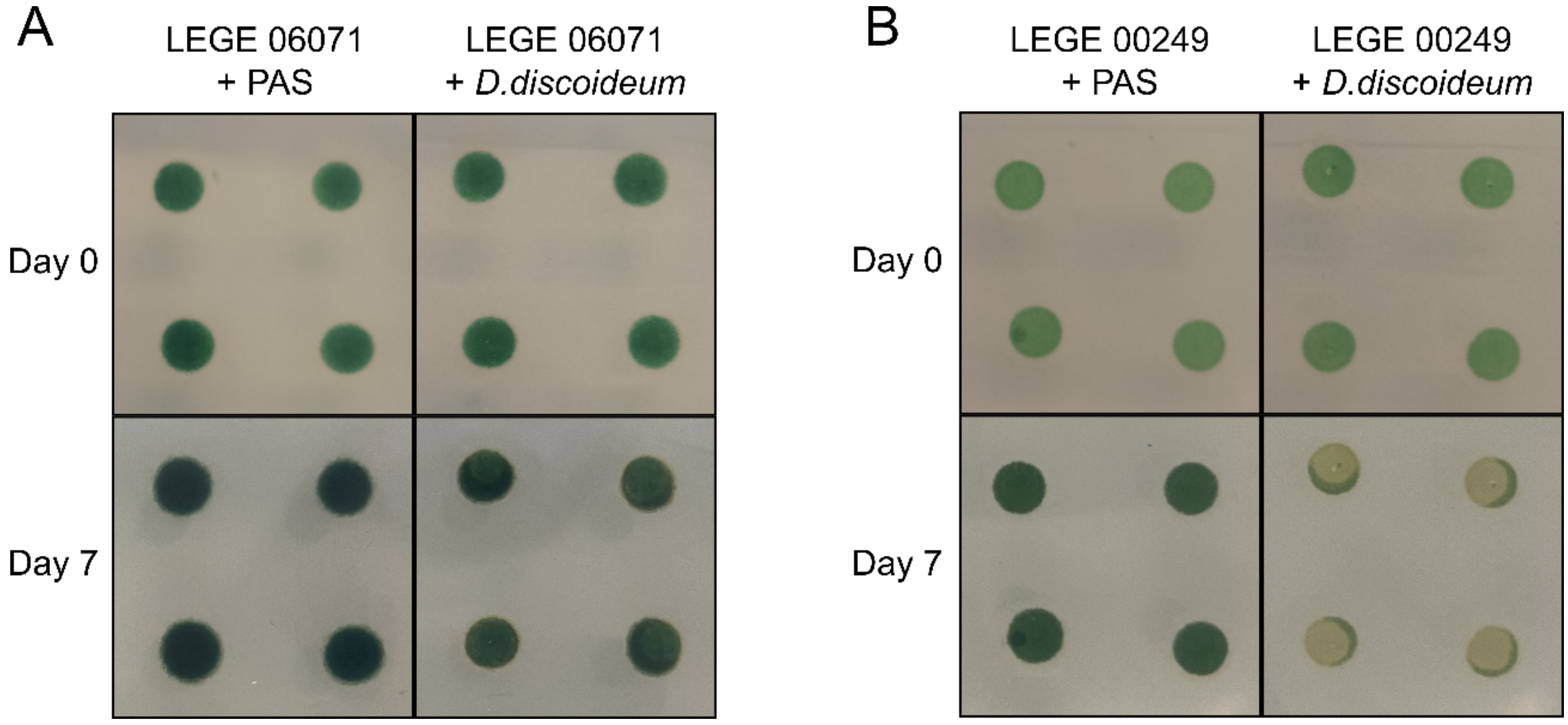

Supplementary Fig. S6: *Nodularia* sp. LEGE 06171 is resistant to *Dictyostelium discoideum* grazing. Cyanobacterial drop grazing assay of A) *Nodularia* sp. LEGE 06071 and B) *Sphaerospermopsis* sp. LEGE 00249 against *D. discoideum* grazing over a period of one week. Yellow areas on top of the co-culture drop indicates grazing of the cyanobacteria. The control drops are cyanobacteria with only PAS amoeba medium on top. This assay was performed in triplicate. *Nodularia* sp. LEGE 06071, the producer strain of nocuolin A, shows resistance to *D. discoideum* grazing, as opposite to *Sphaerospermopsis* sp. LEGE 00249, that is sensitive to this amoebae strain.

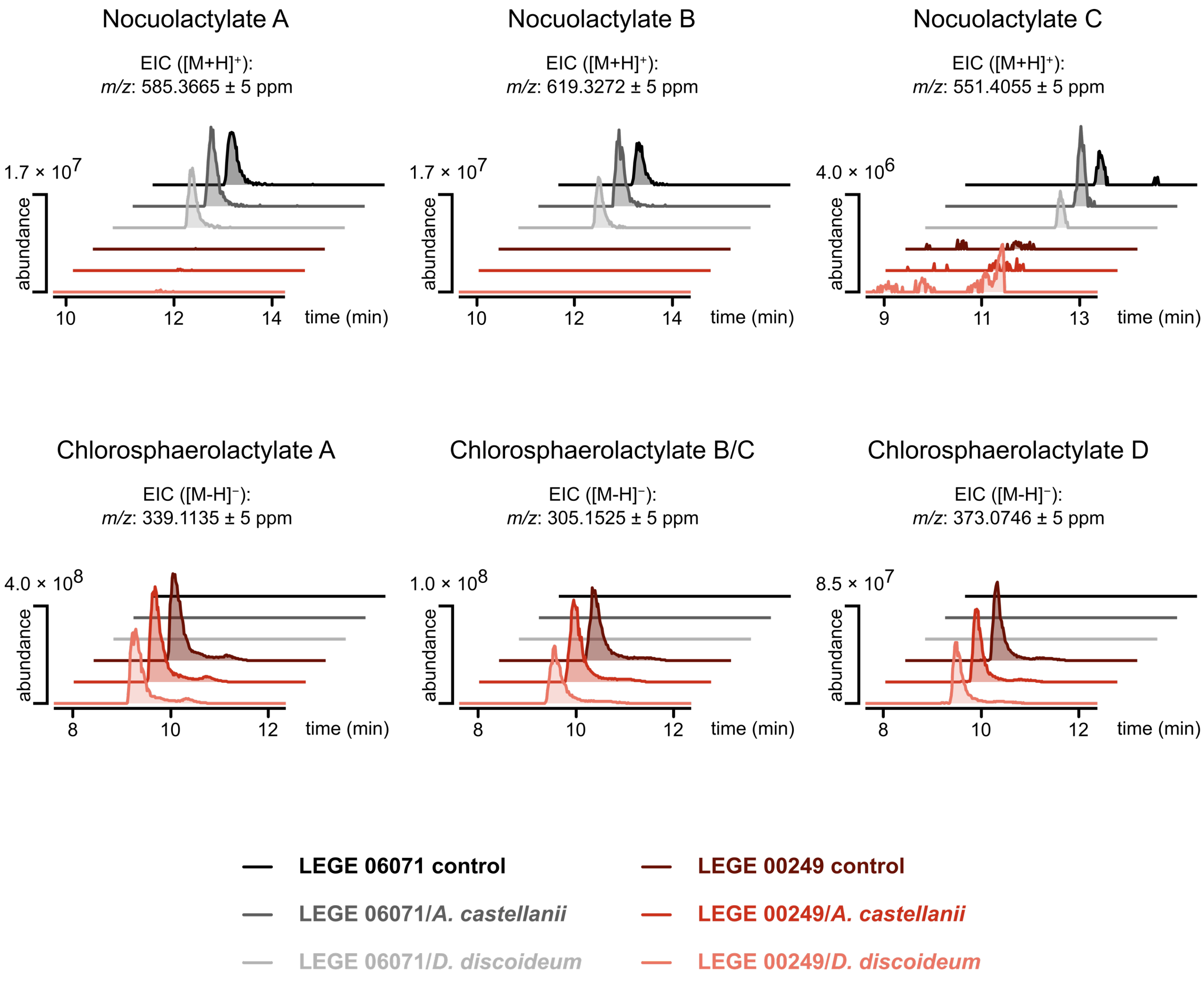

Supplementary Fig. S7: Detection of nocuolactylates and chlorosphaerolactylates in the lawns of the monocultures and co-cultures of *Nodularia* sp. LEGE 06071 and *Sphaerospermopsis* sp. LEGE 00249 with amoebae. LC-HRMS-derived extracted ion chromatograms (EICs) of nocuolactylates A-C detected in the monocultures of LEGE 06071 and its co-cultivation with both *A. castellanii* and *D. discoideum*, and the chlorosphaerolactylates A-D detected in the monocultures of LEGE 00249 and its co-cultivation with *A. castellanii* and *D. discoideum*.

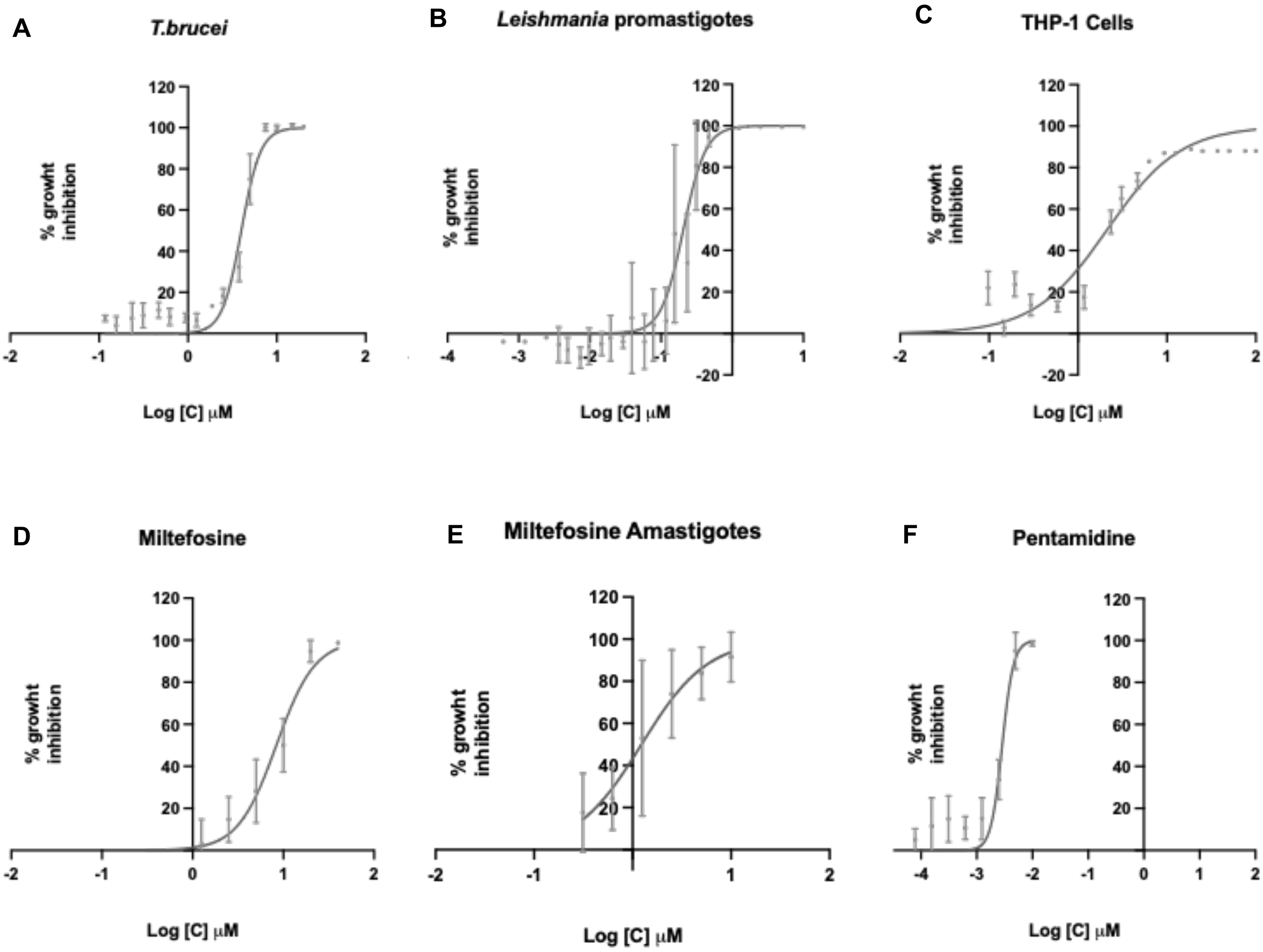

Supplementary Fig. S8: Dose response curves used to generate the IC50 of nocuolin A and the control compounds miltefosine and pentamidine in *Trypanosoma brucei*, *Leishmania infantum* and THP-1 cells. Dose-response curve of nocuolin A in A) *Trypanosoma brucei*, B) *Leishmania infantum* promastigotes, and C) THP-1 cells. Dose-response curve of miltefosine in D) *Leishmania infantum* promastigotes and E) amastigotes and F) pentamidine in *Trypanosoma brucei*. Miltefosine and pentamidine were tested as reference drugs in each correspondent assay. The IC50 for nocuolin A was 3,990 µM, 0,2062 µM and 2,127 µM for *Trypanosoma brucei*, *Leishmania infantum* and THP-1 cells, respectively. The IC50 for miltefosine was 8,369 µM and 2,12 µM for *Leishmania infantum* promastigotes and intracellular amastigotes, respectively and, the IC50 for pentamidine was 0,002864 µM for *Trypanosoma brucei*. Average of three assays.

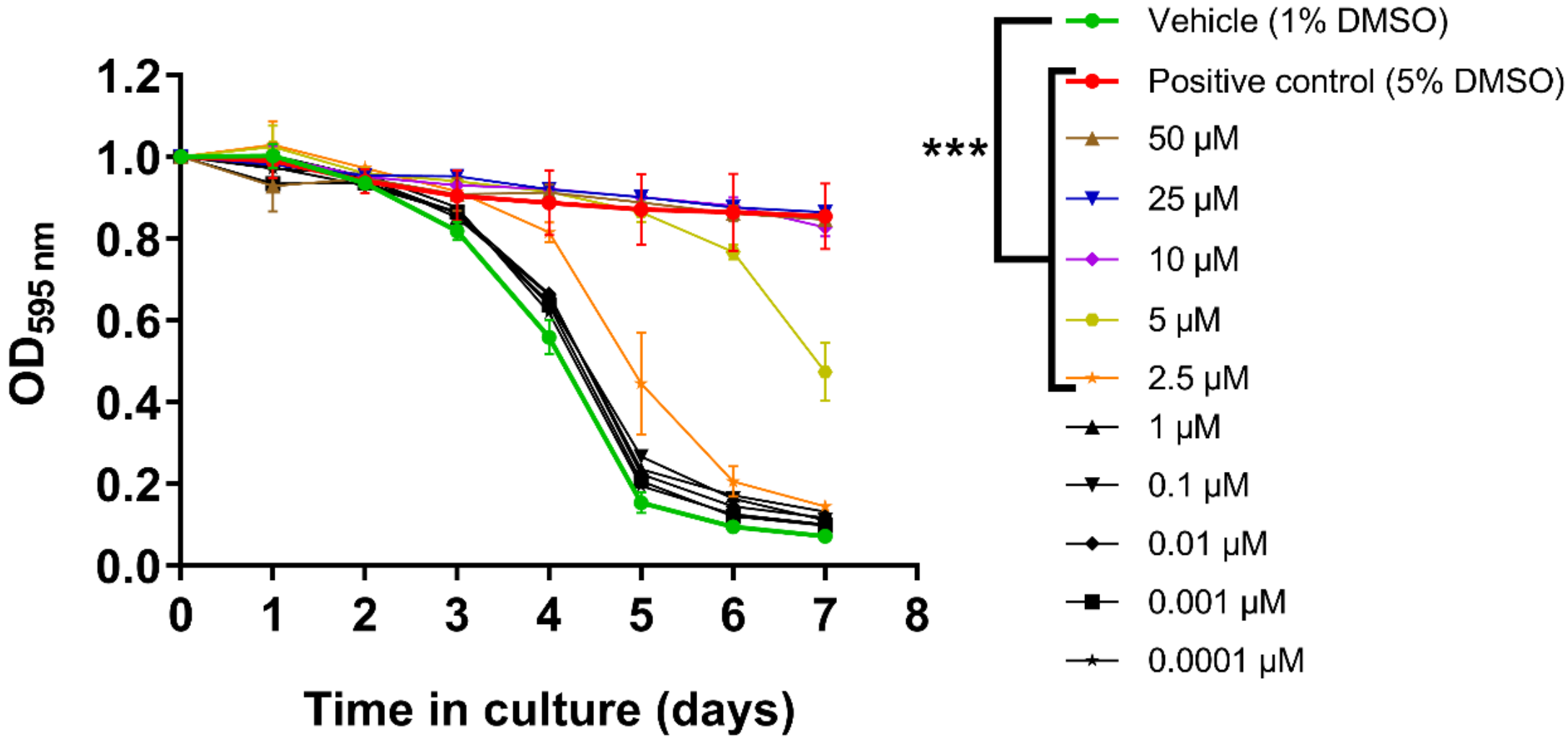

Supplementary Fig. S9: Nocuolin A toxicity evaluation using *C. elegans*. The OD_{595nm} of the *E. coli* OP50 suspension was measured daily of WT animals treated with nocuolin A at the indicated concentrations. For each condition tested, the mean OD_{595nm} was calculated for each day from five replicates and plotted over time. Toxic concentrations of 10 µM and 5 µM was defined as food clearance differs from vehicle (DMSO 1 %). Control DMSO (1 % DMSO) corresponds to drug vehicle and DMSO at 5 % (5 % DMSO) was used as positive (toxic compound) control. WT, wild-type. Average of tree assays.
